# Parasitic and predatory behavior of *Alysia manducator* (Hymenoptera: Braconidae) on blow fly larvae feeding on an adult pig carcass in the Western Cape Province of South Africa: preliminary observations and forensic implications

**DOI:** 10.1007/s00414-023-03001-5

**Published:** 2023-04-21

**Authors:** Adeyemi Daniel Adetimehin, Calvin Gerald Mole, Devin Alexander Finaughty, Marise Heyns

**Affiliations:** 1 Department of Pathology, Division of Forensic Medicine and Toxicology, University of Cape Town, Cape Town, South Africa; 2 Department of Human Biology, Division of Clinical Anatomy and Biological Anthropology, University of Cape Town, Cape Town, South Africa; 3 School of Chemistry and Forensic Science, University of Kent, Canterbury, UK; 4 School of Medicine, Faculty of Life and Health Sciences, Ulster University, Derry/Londonderry, UK

**Keywords:** Wasps, *Alysia manducator*, Parasitism, Decomposition, Post-mortem interval, Forensic entomology

## Abstract

Wasps are part of the entomofauna associated with vertebrate carrion. They are known to parasitize and prey on specific life stages of insect hosts such as eggs, larvae, pupae, and/or adults associated with vertebrate carrion. However, reports of parasitic behavior of wasps on carrion-associated insect life stages and their possible forensic implications are non-existent in the Western Cape Province of South Africa. This study is part of ongoing research investigating the entomofauna and their pattern of succession on an adult pig carcass in Cape Town, South Africa. During this study, the parasitic wasp *Alysia manducator* was noted parasitizing and preying on blow fly larvae associated with the decomposing carcass. The arrival of *A. manducator* coincides with the occurrence of blow fly eggs and/or larvae on the carcass. These wasps were seen in close association with the eggs and larvae of blow flies on various parts of the carcass and some wasps were seen dragging fly larvae attached to their ovipositors away from one part of the carcass to another. Some *A. manducator* were also observed walking over several larvae on the carcass while exhibiting a stabbing behavior presumably in search of a host for oviposition. We suggest that the observations recorded in this study are of considerable forensic importance as the dragging effect and predatory and stabbing behavior exhibited by *A. manducator* could potentially disrupt the feeding and development of the fly larvae on the carcass. This could subsequently alter the process of carcass decomposition and/or affect minimum post-mortem interval estimations.

**Supplementary Information:**

The online version contains supplementary material available at 10.1007/s00414-023-03001-5.

## Introduction

In the forensic entomology literature, attention has mainly been given to insects belonging to the orders Diptera (flies) and Coleoptera (beetles) [1]. This is ostensibly due to their inherent abilities to locate and utilize vertebrate carrion as suitable substrates for feeding and breeding [1–3]. Due to their necrophagic and reproductive tendencies on a vertebrate carrion, researchers have been able to extract valuable information from these insects such as the minimum time since death, time since human/animal neglect, change in cadaver location, and drugs/poisons presence which could further be applied in medico-legal investigations [1].

Other insect orders have, historically, been overlooked. The forensic importance of the order Hymenoptera has recently begun to attract the attention of forensic entomologists. This order comprises insects such as ants which have been documented to not only prey on immature and adult stages of other carrion-associated insects, but also inflict injuries, cause hemorrhage, and prevent fly landing and oviposition on vertebrate remains in various parts of the world [4, 5]. Another interesting insect group within this order is that of the wasps. These insects are frequent responders to decomposing vertebrate remains. Among them is a special group known as parasitoids. These parasitic wasps are necrophilous in nature; however, they do not feed on vertebrate carrion [6]. Instead, they make use of the immature (e.g., eggs, larvae, prepupae, and pupae) stages of blow flies (Diptera: Calliphoridae), flesh flies (Diptera: Sarcophagidae), house flies (Diptera: Muscidae), scuttle flies (Diptera: Phoridae), cheese skippers (Diptera: Piophilidae), and rove beetles (Coleoptera: Staphylinidae) as suitable hosts for the breeding and development of their offspring [6–11].

The parasitic and predatory behavior of wasps have been documented to have substantial implications in biological control programs involving their importation, mass breeding, and deployment in the control and management of invasive and native insect pests and plants in agricultural settings, aquatic environments, urban areas, and animal rearing facilities [12–17]. However, their context in the perspective of forensic entomology has not received the same level of attention. The parasitic and predatory behavior of wasps has been documented to alter the development, morphological appearance, and behavior of the host insect, prevent fly oviposition, prey on adult and immature insect stages, and/or deter the activities of vertebrate scavengers on or around vertebrate carrion [12, 18–23]. This, in turn, may interrupt insect colonization and therefore possibly, the decomposition of the vertebrate carrion. In addition, it can jeopardize the attempt to raise collected immature insect specimens (serving as their hosts) to adults for accurate species identification and/or their application in minimum time since death estimation during forensic investigations [6]. Equally important, wasps can cause post-mortem lacerations and injuries on vertebrate carrion which can potentially create entry sites for other insects and possibly mislead forensic investigators during cause-of-death investigations [21, 24].

Several wasp families have been reported to contain parasitic and predatory species. This includes the families Braconidae, Ichneumonidae, Pteromalidae, Figitidae, Vespidae, Eulophidae, Chalcididae, Diapriidae, Encyrtidae, and Proctotrupidae [11, 20, 24–27]. Depending on the species, parasitic wasps possess the ability to forage and parasitize specific life stages of selected insect hosts and, thus, can serve as alternative forensic indicator species after the consideration of fly evidence in the estimation of the minimum time since death, verification of cadaver relocation, and location of hidden vertebrate remains [6, 19, 28]. The limited availability of published reports on the activities of parasitoid wasps on vertebrate remains alongside their application in forensic entomological investigations might be linked to them being neglected or unseen during forensic entomological investigations due to their small size, tendency to arrive during the mid- to late stages of decomposition, and the paucity of published data on their biology and ecology [6, 26]. Nevertheless, there exist reports on the potential value of parasitic wasps in forensic investigations and minimum time since death estimations [9, 10, 28–30].

In South Africa, parasitoid wasps such as *Nasonia vitripennis* (Hymenoptera: Pteromalidae) and *Trichopria lewisi* (Hymenoptera: Diapriidae) have been found to parasitize the pupae of the forensically important fly, *Chrysomya albiceps* (Diptera: Calliphoridae) in the Kruger National Park of the Limpopo/Mpumalanga Provinces [8]. However, we are not aware of any record of the activities of parasitoid wasps on a vertebrate carrion and carrion-associated immature insects in the Western Cape Province—a locale that is markedly different from a biogeographic perspective to that of the Limpopo and Mpumalanga Provinces across which the Kruger National Park spans. Thus, we report preliminary observations and possible forensic implications of the parasitic and predatory behavior of *A. manducator* (Hymenoptera: Braconidae) on the immature stages (i.e., eggs and larvae) of blow flies associated with a decomposing adult pig carcass in the winter season of the Western Cape Province.

## Materials and methods

### Study site

This study is part of an ongoing winter carrion decomposition and insect successional study within the area of Table Mountain National Park, adjacent to the University of Cape Town Upper Campus, Rondebosch, South Africa (S33°57.682ʹ; E018°27.301ʹ). The Table Mountain National Park is characterized by a rocky terrain situated on the Cape Peninsula, a mountainous region located at the south-western tip of the African continent [5, 31]. The Park is dominated by Mediterranean-like shrubland called fynbos—part of the Cape Floristic Kingdom—which is prone to fire [5, 32]. The park harbors 2285 plant species of which 90 are endemic [5, 32]. Pockets of invasive alien vegetation exist, dominated by *Acacia*, *Pinus*, and *Hakea* species [5, 32].

### Decomposition study and animal model

**Fig. 1:**
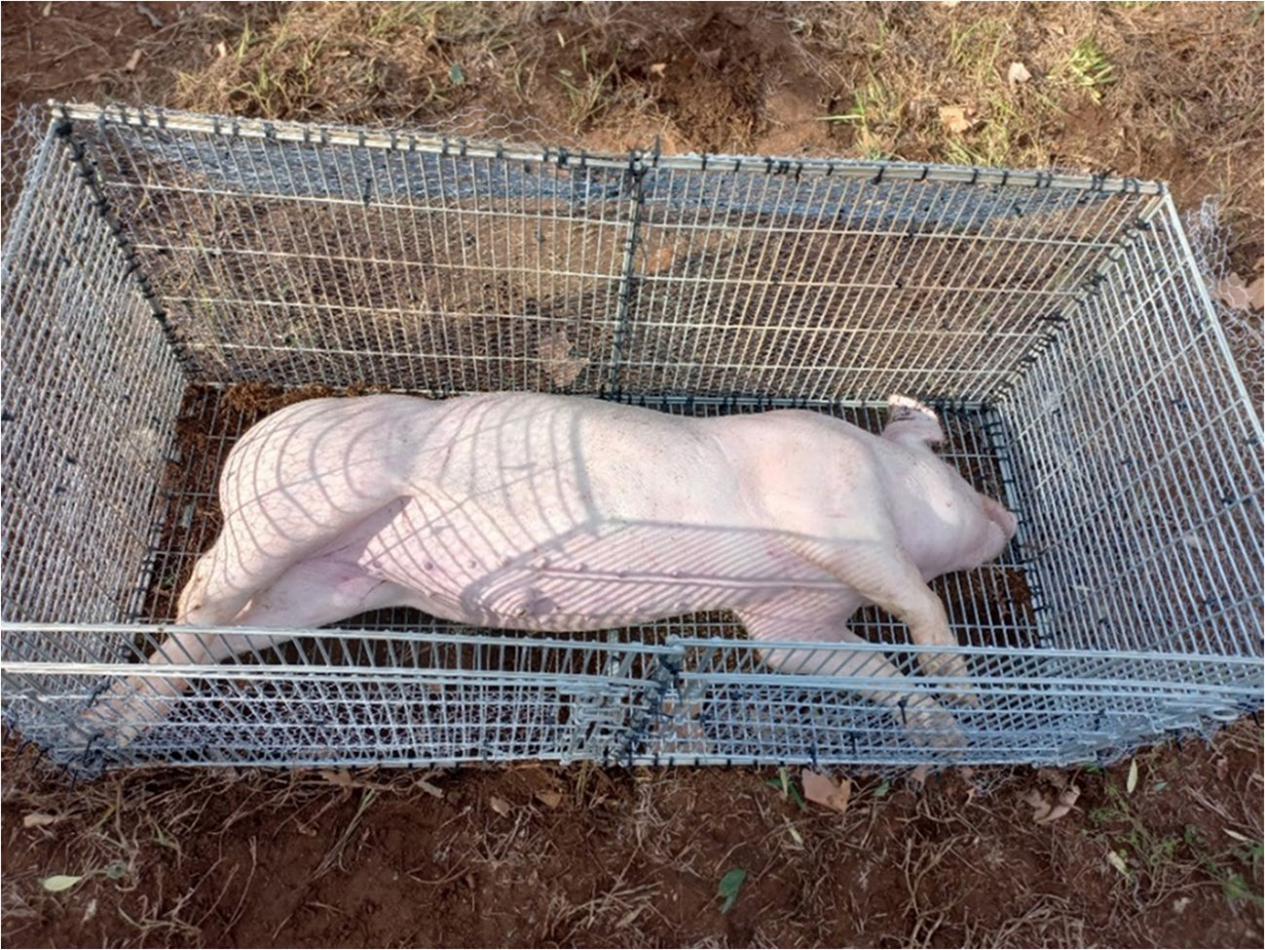
Close-up view of the adult pig carcass within the steel cage

As part of this study, a 60-kg adult pig (*Sus scrofa domesticus* L.) carcass has been used as an animal model. Pigs have been regarded as the most suitable analogues to a human body where the establishment of baseline data is concerned due to the similarities in the integumentary, circulatory, and digestive systems [33]. The adult pig was purchased from the University of Stellenbosch, Faculty of AgriSciences Piggery Unit after which it was humanely terminated by a single shot to the base of the brain with a 0.22 caliber long rifle by the farm manager on the day of the winter solstice (21 June 2022) of the Southern Hemisphere [34]. Immediately following termination, the adult pig carcass was rinsed and sealed within a body bag for transport to the research site. At the research site, the carcass was deployed in a steel cage covered with chicken wire (Fig. 1). Thereafter, decompositional changes and insect activities on the carcass were recorded daily.

### Documentation of observations, collection, and identification of wasps

The location(s) of the wasps on, in, and around the carcass during data collection were documented using detailed field notes and manual photography. The characteristic activities of the wasps on, in, and around the carcass and their interaction with other insect life stages (e.g., fly eggs and larvae) present on and around the carcass were documented with field notes, manual photography, and video recordings. The wasps were collected from the carcass with the aid of soft tweezers after undisturbed video recordings and photographs were taken. After collection, the wasps were transferred immediately into a killing jar containing paper towels dampened with ethyl acetate [5]. Following termination, the wasps were transferred into screwcap containers after which they were taken to the Forensic Entomology Laboratory, Faculty of Health Sciences, University of Cape Town, placed in 70% ethanol, and then stored in a refrigerator until identification.

## Results and discussion

The wasp specimens were identified to be *Alysia manducator* Panzer (Hymenoptera: Braconidae) by Dr. Simon van Noort (Iziko Museums of South Africa). Images of this species and further information is available on WaspWeb [35]. We observed in this study the predatory and parasitic behavior of *A. manducator* on blow fly larvae feeding on animal carrion. The arrival of *A. manducator* individuals coincides with the occurrence of blow fly eggs and larvae on the carcass. We found several individuals of *A. manducator* in close association with the eggs and larvae of blow flies on various parts of the carcass. This observation is consistent with the findings of Horenstein and Salvo [36] in central Argentina and that of our previous studies using neonate pigs in all the seasons of the Western Cape (Adetimehin et al., unpublished data). According to Reznik et al. [19], meat samples infested with *Calliphora vicina* (Diptera: Calliphoridae) larvae were significantly attractive to *A. manducator* when compared to meat samples in similar decomposition stage but without *Ca. vicina* larvae. Similarly, *A. manducator* has been reported to parasitize the larvae of *Lucilia sericata* (Diptera: Calliphoridae) feeding on vertebrate carrion in England, UK [37].

The ability of parasitic wasps (including *A. manducator*) to locate the immature stages of suitable hosts and hosts’ source of nutrition (e.g., vertebrate carrion) might be linked to their sensitivity to the chemical and visual cues of the host insect and its food source [7, 12, 19]. In fact, Heo et al. [38] reported that the parasitoid wasps namely *Exoristobia philippinensis* (Hymenoptera: Encyrtidae) and *Dirhinus himalayanus* (Hymenoptera: Chalcididae) were able to locate and parasitize the pupae of *Liopygia ruficornis* (Diptera: Sarcophagidae) in a baited trap positioned on the rooftop of a high-rise building approximately 101 m above the ground. The forensic application of this is that the foraging behavior of wasps and their ability to detect the chemical cues of their host insect and its food source can serve as useful alternative pointers to the presence of decomposing vertebrate remains and other carrion-associated immature insect life stages (e.g., larvae) in concealed environments (e.g., in the bushveld) and multistorey buildings. Advances in biosensor research have demonstrated the use of wasp species as biological sensors in the identification or detection of specific visual, chemical, and odor cues. Particularly, this has led to the development of the “wasp hound”, a device originally used to detect volatile fungal chemicals [39]. Further research has indicated the ability of conditioning wasp species to detect certain odors or chemical cues associated with animal food products [40], explosives [41, 42], fungal pathogens [43], illicit drugs [44], and vertebrate carrion [45]. Based on the current observations, we suggest that further studies within the region should utilize the “wasp hound” in the training and conditioning of adult *A. manducator* individuals specifically in the detection of the volatile organic chemicals (VOCs) emanating from vertebrate carrion, alongside carrion-associated insects, and the vertebrate carrion itself in different depositional environments (e.g., above ground, hanged, buried, vehicular confinement, trashcan, suitcase, and drum).

### Parasitic behavior: stabbing of fly larvae

**Fig. 2:**
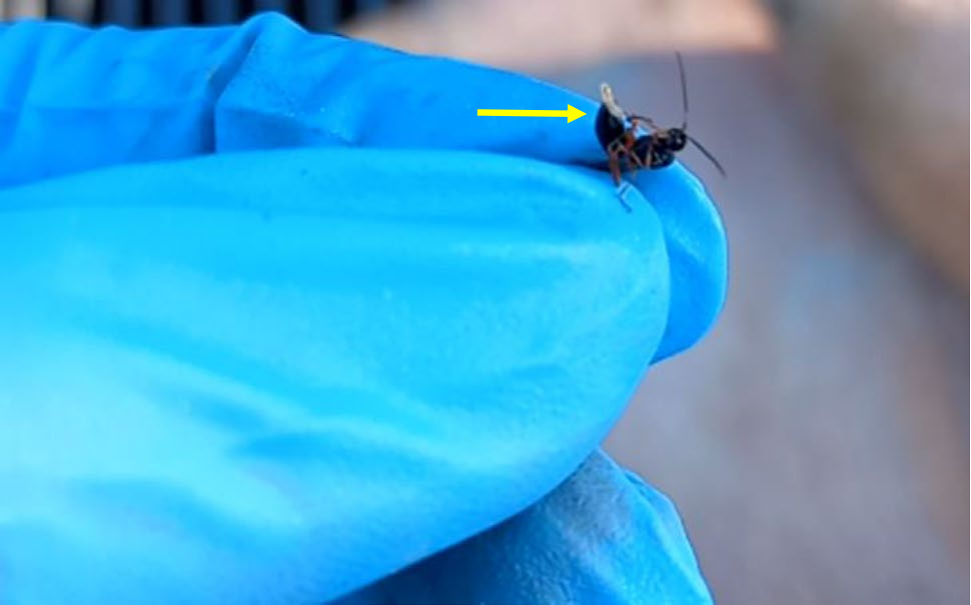
A blow fly larva attached to the ovipositor (yellow arrow) of a collected *Alysia manducator* individual

We observed some individuals of *A. manducator* walking over several larvae on the carcass and then exhibiting a stabbing behavior (Online Resources 1 and 2) presumably in search of a location or host for the purpose of egg laying [7, 11, 19, 46, 47]. This was subsequently confirmed by the presence of a fly larva attached to the ovipositor of one of the collected wasp individuals (Fig. 2). We attribute the commencement of the stabbing behavior by *A. manducator* to the concentration of the decay odor emanating from the carcass and the movement of the fly larvae underneath its legs [7, 19]. Parasitic wasps including *A. manducator* have been reported to exhibit similar behavior on vertebrate carrion [7, 11, 19]. After the stabbing of the host insect, the cuticular teeth on the ovipositor of the parasitoid wasp pierces and ruptures the host insect’s skin [46, 47]. Furthermore, the stabbing behavior of parasitic wasps (including *A. manducator*) brings about the paralysis of the host insect larvae [19, 47]. Owing to this, we speculate that the observed stabbing of the blow fly larvae by *A. manducator* could potentially cause injuries/damages to the soft tissues of the blow fly larvae.

Furthermore, the stabbing behavior of parasitic wasps is a mechanism associated with oviposition [19], and after oviposition/egg eclosion, the likely consumption of the tissues of the immature host insect (e.g., pupa) by the larval stage of the parasitoid wasp could lead to the death of the insect host [18]. In addition, the subsequent development and emergence of the adult parasitoids from their host insects could compromise the morphology and rearing process of collected immature insect specimens (e.g., larvae/pupae) to adults for species confirmation during crime scene investigations. A notable study in this regard was that of Heo et al. [20]. The authors reported the presence of two exit holes on the pupal casing of *Chrysomya rufifacies* (Diptera: Calliphoridae) following the emergence of the adults of the parasitoid wasp, *E. philippinensis*. Equally important, the stabbing behavior by wasps could alter the physiology (e.g., diapause) and development of the immature insect hosts, and manipulate their biology (e.g., feeding) and behavior (e.g., movement) [12, 18, 48]. For example, Cammack et al. [12] reported that the presence of *N. vitripennis* females led to an increase in the developmental rate of *L. sericata* pupae. Similarly, parasitism by *A. manducator* triggered premature pupation in *L. sericata* [48]. Furthermore, Cammack et al. [12] revealed that *L. sericata* larvae burrowed into the soil to escape parasitism by *N. vitripennis*. The resulting impacts of the stabbing behavior by *A. manducator* are substantial and from the forensic entomological point of view, it can potentially influence the estimation off the age of immature fly specimens and their utilization in minimum time since death estimations. Thus, we suggest that forensic entomologists consider the presence/absence of wasps in, on, or around vertebrate remains prior to and after the collection of immature entomological evidence before their utilization in minimum time since death estimations.

### Predation and dragging of fly larvae

**Fig. 3:**
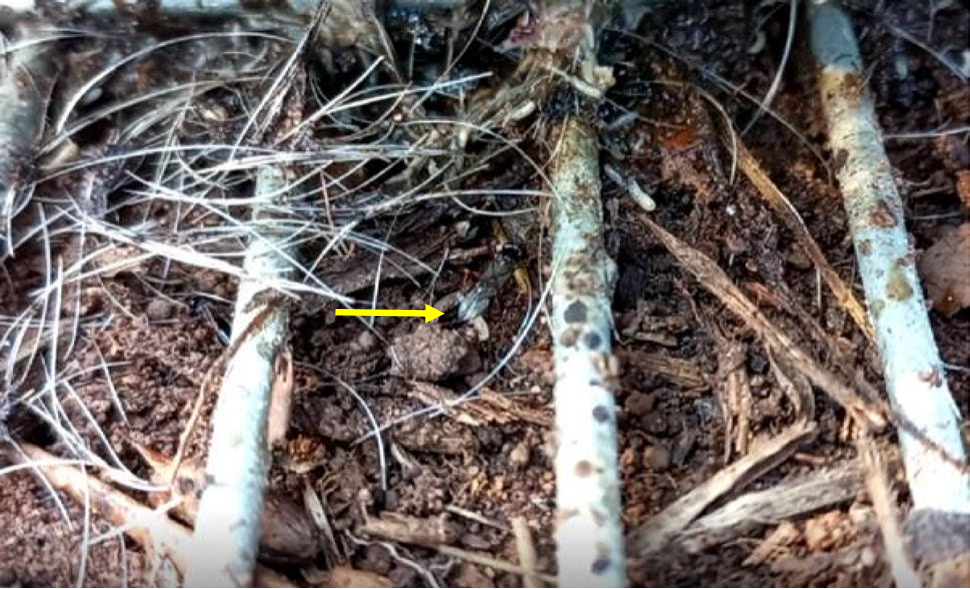
An *Alysia manducator* individual on the soil surface dragging a blow fly larva attached to its ovipositor (yellow arrow)

We found one *A. manducator* individual close to the oral cavity preying on a blow fly larva (Online Resource 3). Some individuals of *A. manducator* were also seen dragging away blow fly larvae attached to their ovipositors (Fig. 3; Online Resources 4 and 5). The ability of *A. manducator* to drag or pull away the immature stages of flies from or toward the carcass or from one body part to another can potentially alter the process of decomposition. For instance, the dragging of a fly larva that has successfully established an entry point or close to establishing one on the skin of a carcass to another region of the body that has not been impacted, can potentially increase the feeding time the specific larva has to go through to gain access to the soft tissues and internal organs of the body.

The persistence of the ovipositor and the continuous dragging of the blow fly larvae by *A. manducator* could potentially overdose the host insect with more toxins and may bring about the premature death of the host larvae [47]. Also, it should be pointed out that the persistence of the wasp’s ovipositor in the blow fly larva and the resultant dragging could be as a result of the level of experience of the female parasitoid wasp in egg laying [47]. In the literature, some studies have documented how parasitoid wasps drag or pull away their host insects with the aid of their ovipositors and mandibles [49, 50]. While the structure of the ovipositors in parasitic Hymenopterans are similar, parasitoid wasps such *Leptopinila heterotoma* (Diptera: Hymenoptera) have been reported to possess a structure called the “ovipositor clip” in their ovipositors which play a key role in holding their hosts [47].

### Present but exhibiting no observable parasitic and/or predatory behavior

Interestingly, for some *A. manducator* individuals, we did not observe them exhibiting any stabbing and/or predatory behavior on or toward the blow fly larvae. Although *A. manducator* individuals are known to be parasitoids of the larvae of flies associated with vertebrate remains [7], their behavioral variation as observed in this study might be due to the age and size of the fly larvae, maturity status and oviposition experience of the female wasp, parasitism status of the fly larvae, and/or odor trail left behind on/around the site of oviposition by the host parent [7, 19, 47, 51]. According to Reznik et al. [19], the increase in probing/contact ratio of *A. manducator* on *Ca. vicina* larvae, for example, may be attributed to the increase in size of the larvae within the 5-day developmental period. However, the possible increase in strength of the larvae due to the size increase may have contributed to the reduction in the oviposition and attack of *A. manducator* on *Ca. vicina* larvae (Reznik et al. [19]). Furthermore, the authors revealed that the decrease in the probing behavior of *A. manducator* on mature *Ca. vicina* larvae could be as a result of the reduction in the smell of the larvae as evidenced by their empty intestinal contents.

### Future research

The current study presents observations on the predatory and parasitic behavior of the wasp *A. manducator*, highlighting the need for further research to ascertain the degree of effect such species may have on the estimation of the minimum time since death. While previous research has demonstrated developmental, morphological, physiological, and behavioral changes in host species of parasitoid wasps [12, 18–20, 48], further laboratory-based research is needed to fully understand the medico-legal implications of such behavior. The use of wasp species in the detection of human remains is a further avenue of interest, as advances in the literature have demonstrated the effectiveness of a device called the “wasp hound” in the training and conditioning of wasp species as biological sensors in the identification or detection of specific visual, chemical, and odor cues associated with food products, explosives, illicit drugs, and vertebrate carrion [40–45].

## Conclusion

The current study presents observations on the predatory and parasitic behavior of the wasp *A. manducator* associated with a decomposing adult pig carcass. While much of the observations have previously been reported in ecological and entomological literature, the current study highlights the importance of these observations for forensic entomology.

We observed *A. manducator* stabbing the larvae of blow flies on multiple occasions. Also, we noted the dragging of some larvae away and/or toward the carcass. We speculate that the (1) stabbing behavior of *A. manducator* can cause injuries to the soft tissues of the larvae which can alter the development and behavior of blow fly larvae and their utilization in minimum time since death estimations; (2) dragging of blow fly larvae away from the carcass or from one part of the carcass to another can influence the rate of decomposition of a vertebrate carrion particularly in the early stages where minimal larvae are present; (3) likely oviposition of the wasps and the development of their offspring on/in the larvae can potentially inhibit the development of the collected immature insects to adults for accurate species identification; and (4) ability of wasps to locate their insect host and its associated food source can provide useful indications as to the location of cadavers and cadaver-associated immature insects in concealed environments.

These observations highlight the need for further research to ascertain the degree of effect such species may have on the estimation of the minimum time since death. Particularly, there is a need for more laboratory and field studies on the biology, ecology, and foraging behavior of *A. manducator* on food resources including vertebrate carcasses in the presence or absence of the immature stages (e.g., eggs, larvae, and pupae) of similar/multiple forensically important fly species. This may help clarify if *A. manducator* exhibit any host specificity in relation to its parasitic behavior toward larvae of similar/multiple fly species and how such behavior affects the larvae’s feeding and development. We also suggest the need for laboratory and field studies focused on examining the effectiveness of trained/conditioned adult *A. manducator* individuals in the detection of vertebrate carrion and carrion-associated chemical cues in our region. Lastly, as with other similarly cited articles from different parts of the world, we urge forensic entomologists to take cognizance of the presence or absence of wasps in/around the crime/death scene during entomological evidence collection as this may have an influence on the development of the insect evidence prior to or after collection and their utilization in minimum post-mortem interval estimations.

### Supplementary Information

Below is the link to the electronic supplementary material.

Supplementary file1 (MP4 30712 KB)

Supplementary file2 (MP4 23883 KB)

Supplementary file3 (MP4 8444 KB)

Supplementary file4 (MP4 15788 KB)

Supplementary file5 (MP4 16445 KB)

## Data Availability

All data generated or analyzed in relation to this study are included in this published article and its supplementary information files.
